# Anti-VEGF treatment for acute ROP – *not yet recommended!*

**Published:** 2014

**Authors:** Clare Gilbert

**Affiliations:** Co-director: International Centre for Eye Health, Disability Group, London School of Hygiene and Tropical Medicine, London, UK.

**Figure:**
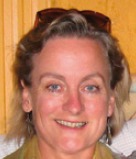
Clare Gilbert

There are several reasons why anti-VEGF agents are not recommended for acute, severe retinopathy of prematurity (ROP).

- There has only been one randomised trial, which compared laser with Avastin (bevacizumab) for Type 1 ROP. It was only more effective in preventing early recurrence of severe disease in Zone 1 (posterior), but the recurrence rate in the laser arm was worse than would be expected based on other studies. More babies died in the anti-VEGF arm of the trial, but the difference was not statistically significant.
- There are major concerns about the short- and long-term impact of anti-VEGF agents on the lung, kidneys and brain of a baby.
- Follow-up studies, using fluorescein angiography, indicate that normal retinal vascularisation may not take place after administration of bevacizumab, with extensive areas of non-perfusion months after treatment.
- There are an increasing number of case reports which show that, although Avastin can lead to regression of ROP in the short term, the ROP can recur months later. This means that an acute disease with a known natural history has the potential to become a chronic disease with an unknown and unpredictable natural history.
- Some surgeons use Avastin for Stage 4a or 4b ROP prior to surgery. This can make the surgery easier, but there is still the risk of systemic complications.

The leaking capillaries present in eyes with retinopathy increase the risk of large molecules (e.g. anti-VEGF drugs injected into the eye) entering the systemic circulation, and so the systemic safety of these drugs is important, particularly in preterm infants. If anti-VEGF drugs seem to be the only option to preserve sight when extensive laser has failed, or the infant is too sick for laser, this treatment can be offered, but **only** after parents have been fully informed of the possible consequences.

